# Potent Application of Scrap from the Modified Natural Rubber Production as Oil Absorbent

**DOI:** 10.3390/polym14235066

**Published:** 2022-11-22

**Authors:** Anoma Thitithammawong, Sitisaiyidah Saiwari, Subhan Salaeh, Nabil Hayeemasae

**Affiliations:** 1 Research Unit of Advanced Elastomeric Materials and Innovations for BCG Economy (AEMI), Faculty of Science and Technology, Prince of Songkla University, Pattani Campus, Pattani 94000, Thailand; 2 Department of Rubber Technology and Polymer Science, Faculty of Science and Technology, Prince of Songkla University, Pattani Campus, Pattani 94000, Thailand

**Keywords:** natural rubber, scrap rubber, oil absorbency, foam, waste

## Abstract

The production of raw natural rubber always ends up with leftover latex. This latex is later collected to produce low grades of rubber. The collection of this latex also depends on the latex’s quality. However, reproducing the latex may not be applicable if the latex contains many specks of dirt which will eventually be discarded. In this work, an alternative solution was to utilize such rubber in a processable form. This scrap rubber (SR) from the production of natural rubber grafted with polymethyl methacrylate (NR-g-PMMA) production was recovered to prepare an oil-swellable rubber. The rubber blends were turned into cellular structures to increase the oil swellability. To find the suitable formulation and cellular structure of the foam, the foams were prepared by blending SR with virgin natural rubber (NR) at various ratios, namely 0/100, 20/80, 30/70, 50/50, 70/30, 80/20, and 100/0 (phr/phr). The foam formation strongly depended on the SR, as it prevented gas penetration throughout the matrix. Consequently, small cells and thick cell walls were observed. This structure reduced the oil swellability from 7.09 g/g to 5.02 g/g. However, it is interesting to highlight that the thermal stability of the foam increased over the addition of SR, which is likely due to the higher thermal stability of the NR-g-PMMA waste or SR. In summary, the blending NR with 30 phr of SR provided good oil swellability, processability, and morphology, which benefit oil recovery application. The results obtained from this study will be used for further experiments on the enhancement of oil absorbency by applying other key factors. This work is considered a good initiative for preparing the oil-absorbent material based on scrap from modified natural rubber production.

## 1. Introduction

Oil spills in the sea have become a crucial problem for marine environments. It is turning into a more significant issue due to the expansion of the offshore oil sector and the requirement for marine oil transportation [1,2,3]. This is also not limited to an oil spill in industries or workshops, especially from the machinery and piping systems. In the past, different techniques have been used to clean up oil spills, including in situ burning, mechanical collection of chemical dispersants, bioremediation, and the use of absorbent materials [4,5]. Owing to the economy and efficiency of oil collection and remediation, searching the compromising methods is the most desirable choice. Therefore, it is essential to look for a practical means of producing an absorbent substance for oil cleanup.

Various polymers have been widely used for oil spill redemption. Among them, alkyl acrylate and its derivatives, which have high hydrophobicity, have been attracting much interest in oil spill redemption. Jadhav et al. [6] prepared graft copolymerization of Poly(methyl methacrylate) (PMMA) on the backbone of *Meizotropis pellita* fibers (MPF). A PMMA-grafted MPF by 120% absorbed crude oil at 23.60 g, 17.41 g, and 14.21 g for the first to the third cycle of absorption, whereas diesel oil was absorbed from 13.14 g to 5.25 g for three cycles of absorption. Grafted PMMA onto natural rubber (NR) foam for oil sorbent was prepared by Ratcha et al. [7]. It was claimed that the modified NR foam could rapidly absorb gasoline and petroleum-derived organic solvents like toluene and xylene. The maximum oil absorbency of gasoline, diesel, engine oil, toluene, and xylene was achieved at 9.95 g/g, 8.37 g/g, 6.01 g/g, 11.81 g/g, and 10.96 g/g, respectively. Kanagaraj et al. [8] also prepared the MMA-grafted natural rubber foam with varying PMMA percentages, i.e., 0%, 10%, 20%, 30%, 40%, and 50%. The hardness, compression set, and stiffness of the natural rubber foam increased as the percentage of PMMA increased. The addition of MMA tended to swell more when soaked in toluene.

As stated, PMMA-grafted NR was previously applied for oil-absorbent material and found to be increased over the grafting content of PMMA onto natural rubber. PMMA-grafted NR has been studied and prepared in the last few decades. In the presence of MMA, the polarity of NR increases and can be extensively used in adhesive applications. During the synthesis of NR-g-PMMA, there is abundant rubber waste caught at the end of the manufacturing stage that is particularly not further processed as it contains many specks of dirt. The idea of this work was to utilize the waste as a blending component with NR to prepare oil-absorbent materials based on the scraps from the production of NR-g-PMMA. As previously mentioned, the acrylate-type polymer has been utilized as an oil-absorbent material. It is expected that the NR-g-PMMA could also apply the same solution.

To increase the oil absorbency of the rubber, the appearance of the rubber should be modified by turning the general solid form of rubber into a cellular structure. Cellular rubber, or so-called rubber foam, is an interesting rubber product that consists of rubber and gas phases in one item. Natural rubber foam is lightweight, has excellent thermal insulation, and absorbs sound, making it a popular choice for various applications [9,10]. Further, the porosity of rubber can also increase the penetration efficiency of oil towards the rubber. There are several methods to produce rubber foam, either latex or dry stages. As the collected scraps from the final production are in solid form, it is better to prepare the foam using an additive called a blowing agent. The foam structure can be controlled by the proper selection of blowing agents and curatives, which achieves the correct balance between the gas generated and the degree of curing. There are many types of chemical blowing agents, such as azodicarbonamide (ADC), dinitroso pentamethylene tetramine (DPT), sodium bicarbonate, p-toluenesulphonyl semicarbazide, 5-phenyl tetrazole, and 4,4-oxydibenzenesulphonyl hydrazide (OBSH). Numerous authors have investigated how elastomers are influenced by the content and type of a chemical blowing agent [11,12,13]. ADC is an attractive chemical blowing agent among these since it produces uniform cell foam and decomposes at low temperatures [14].

This work is a preliminary study on the potential application of scrap rubber (SR) as an oil absorbent material. Here, various blending ratios between NR and SR were designed and their sorption capacities, mainly for crude oil solution, were determined. The results obtained from this study will be used for further experiments on the enhancement of oil absorbency by applying other key factors. This is considered a good initiative for preparing the oil-absorbent material based on scraps from raw rubber production.

## 2. Experimental Details

### 2.1. Materials

The natural rubber (NR) used in this study was STR 5L, purchased from Suansom Kanyang, Yala, Thailand. Scrap rubber (SR) was supplied by a local company in the Southern part of Thailand. It was from the production of NR-g-PMMA in their factory. The basic properties of NR and SR are listed in Table 1. It can be seen that the properties were more or less the same except for the acetone extract, original plasticity (P_o_), and Mooney viscosity (MU). The higher acetone extract was due to the unreacted chemicals left after the production of SR whereas higher P_o_ and MU were due to the PMMA hard phase that might affect the hardness and the viscosity of the SR. ADC was used as the blowing agent and was purchased from A.F. Supercell Co., Ltd., Rayong, Thailand. Treated distillate aromatics extract (TDAE oil) was used as processing oil. Processing oil can help to reduce the viscosity and facilitate the diffusion of gas [15]. TDAE oil was purchased from H&R ChemPharm (Thailand) Co., Ltd. Calcium carbonate (CaCO_3_) was obtained from Krungthepchemi Co., Ltd., Bangkok, Thailand. It is widely known to act as a nucleating agent in the production of foam. Stearic acid was used as a curing activator and was purchased from Imperial Chemical Co., Ltd., Bangkok, Thailand. ZnO was used as a curing activator and foam kicker which was obtained from Global Chemical Co., Ltd., Samut Prakan, Thailand. 2,2,4-trimethyl-1,2-dihydroquinoline (TMQ) and N-cyclohexyl-2-benzo thiazole sulphenamide (CBS) were used as an antioxidant and accelerator, respectively. These were bought from Flexsys America L.P., West Virginia, USA. Sulfur, which was used as a curing agent, was purchased from Siam Chemical Co., Ltd., Samut Prakan, Thailand.

### 2.2. Preparation of the Rubber Foams

Table 2 lists the materials and mixing sequence for compounding the oil-absorbent foam. SR and NR ratios were 0/100, 20/80, 30/70, 50/50, 70/30, 80/20, and 100/0 (phr/phr), respectively. The full amounts of the SR or NR, ADC, TDAE oil, and other additives were prepared using a two-roll mill. Proper control of Mooney viscosity was encouraged for preparing the rubber foam. The compounds were free-blown into specific shapes (12.5 mm in thickness) inside the compression-molded at the temperature of 150 °C. The time consumed was based on the curing times measured by a moving-die rheometer (MDR), as described in the following section.

### 2.3. Measurement of Curing Characteristics

Using an MDR (Rheoline, Mini MDR Lite) at 150 °C, the curing characteristics of the rubber were measured according to ASTM D5289. This was utilized to calculate the torque, scorch time (ts_1_), and cure time (tc_90_).

### 2.4. Measurement of Relative Foam Density and Expansion Ration

The physical properties were investigated, including the relative foam density and expansion ratio. The relative foam density was measured according to ASTM D3575, using Equation (1) as given below.

Relative foam density = *D_f_/D_c_* (1)

where *D_f_* is foam density (g/cm^3^), and *D_c_* (g/cm^3^) is compound density.

Equation (2) illustrates how the expansion ratio was calculated by comparing the density of the natural rubber compound specimen to the density of the natural rubber foam.

Expansion ratio = *D_c_/D_f_* (2)

### 2.5. Measurement of Hardness

The hardness of the specimens was measured in accordance with ASTM D2240 by using an indentation durometer shore OO, and the readings were taken after a 10-second indentation.

### 2.6. Oil Absorbency

The sample was prepared by cutting into a dimension of 1.5 × 1.5 × 1.0 cm^3^. Then, the sample was weighed prior to immersion in crude oil solution. The swelling lasted for a certain period of time, where the development of swelling uptake or oil absorbency was measured to find the equilibrium swelling. To measure the swollen sample, the sample was tapped with filter paper to remove excess oil and then weighed on a balance. The oil absorbency was calculated by the following formula.

Oil absorbency = *W_a_*/*W_b_* (3)

where *W_a_* is the weight of absorbed oil, and *W_b_* is the weight of the sample. The unit of oil absorbency is g/g which is the mass of oil swells per 1 g of sample.

### 2.7. Diffusion Studies

Diffusion studies were carried out simultaneously as the oil absorbency test but calculated differently. The test piece was weighed using a series of time intervals, beginning with 10 min for the 1^st^ hour, followed by 20 min for the 2^nd^ hour, 30 min for the 3^rd^ hour, and then hourly until the test piece’s weight reached equilibrium. The solvent uptake was employed to create a plot of the sorption curve with the mole percent (mol%) against the square root of the time (*t*^1/2^). The equation is displayed as follows.

(4) $$Q_{t}=\frac{\left(\frac{m_{t}-m_{o}}{M_{w}}\right)}{m_{o}}\times 100$$

where *Q_t_* is the mole percent uptake, *m_t_* is the weight of the swollen sample at a given time, *m_o_* is the initial weight of the sample, and *M_w_* is the molecular weight of the solvent.

The swelling coefficient, which represents the sample’s swelling behavior, can then be calculated by inserting the weight into Equation (5).

(5) $$\beta =\frac{m_{\infty }-m_{o}}{m_{o}}\times \rho_{s}{}^{-1}$$

where *β* denotes the swelling coefficient, *m_∞_* is the weight of the sample at equilibrium, *m_o_* is the weight of the test piece before swelling, and *ρ_s_* is the solvent’s density.

Diffusivity (*D*) is a kinetic parameter that depends on segmental polymer mobility. *D* can be calculated using Equation (6) (the second Fickian law):

(6) $$D=\pi {\left(\frac{h\theta }{4Q_{\infty }}\right)}^{2}$$

where *h* is the thickness of the sample before swelling, *θ* is the slope of the linear sorption curve, and *Q_∞_* is the equilibrium solvent uptake.

The action of permeate molecules initially penetrating and dispersing within the polymer matrix is explained by sorption. Equation (7) can be used to calculate the sorption coefficient from the swelling.

(7) $$S=\frac{m_{\infty }}{m_{o}}$$

where *m_∞_* is the weight of the solvent taken at equilibrium and *m_o_* is the initial mass of the polymer sample.

Equation (8) can be used to estimate the permeability coefficient (*P*), which provides data on how much solvent permeates through a uniform region of the sample each minute.

(8) P=DS

Yao et al. [16] proposed a practical technique for determining how much solution is released from a slab of time (*t*) which is in terms of the total amount of solvent uptake as shown in Equation (9).

(9) $$\frac{Q_{t}}{Q_{\infty }}=kt^{n}$$

The sorption curve’s result can be fitted to the empirical data to determine the samples’ mode of transport, as indicated in Equation (10).

(10) $$\log\frac{Q_{t}}{Q_{\infty }}=\log k+n\log t$$

where *Q_t_* is the mole percent uptake, *Q_∞_* is the equilibrium solvent uptake, *k* is the constant, all of which depend on the polymer’s structural properties and how the sample interacts with the solvent. The magnitude of *n* indicates the type of transport. The linear regression was used to calculate the values of *n* and *k*.

### 2.8. Optical Image and Scanning Electron Microscopy

The physical appearance of natural rubber foams was captured using a mobile phone camera through a default setting. The morphology was screened using a FEI Quanta™ 400 FEG scanning electron microscope (SEM; Thermo Fisher Scientific, Waltham, MA, USA). Each specimen was coated with a layer of gold/palladium to remove the charges that had built up during imaging.

### 2.9. Thermogravimetric Analysis (TGA)

A PerkinElmer Pyris 6 TGA analyzer was used to perform a thermogravimetric analysis on the samples. The sample was heated at a rate of 10 °C/min in a nitrogen flow while being scanned from 30 °C to 600 °C.

### 2.10. Fourier Transform Infrared-Spectroscopic Analysis (FT-IR)

Fourier transform infrared spectroscopy (FTIR) was used to examine the functionalities shown in NR and SR via the FTIR spectroscope model TENSOR27 (Bruker Corporation, Billerica, MA, USA). The spectra were captured in transmission mode throughout the range of 4000–550 cm^−1^ at a resolution of 4 cm^−1^.

## 3. Results and Discussion

### 3.1. Characterization of NR and SR

**Figure 1:**
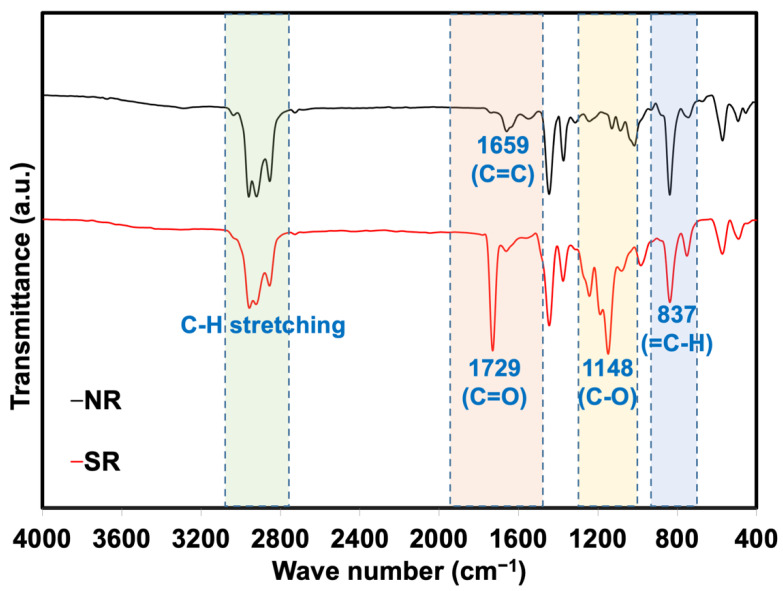
FTIR spectra of NR and SR.

Figure 1 shows the FTIR spectra of NR and SR. These two infrared spectra were different: SR shows intense absorption peaks at 1729 cm^−1^ and 1148 cm^−1^, which are associated with -C=O and -C-O groups in the PMMA chains grafted onto the NR molecules. The absorbance ratios of the peaks at 1729 cm^−1^ to 837 cm^−1^ might be used to roughly evaluate the amount of grafted PMMA on the NR molecules. The 837 cm^−1^ peak results from the =C-H out-of-plane bending of cis-1,4 polyisoprene, whereas the 1729 cm^−1^ peak is related to the C=O stretching of grafted PMMA. The result clearly shows a higher intensity of 1729 cm^−1^ over the 837 cm^−1^, suggesting that higher PMMA was grafted to NR molecules. The peaks observed in SR agreed well with the previous works on the preparation of PMMA grafted onto NR molecules [17,18,19].

**Figure 2:**
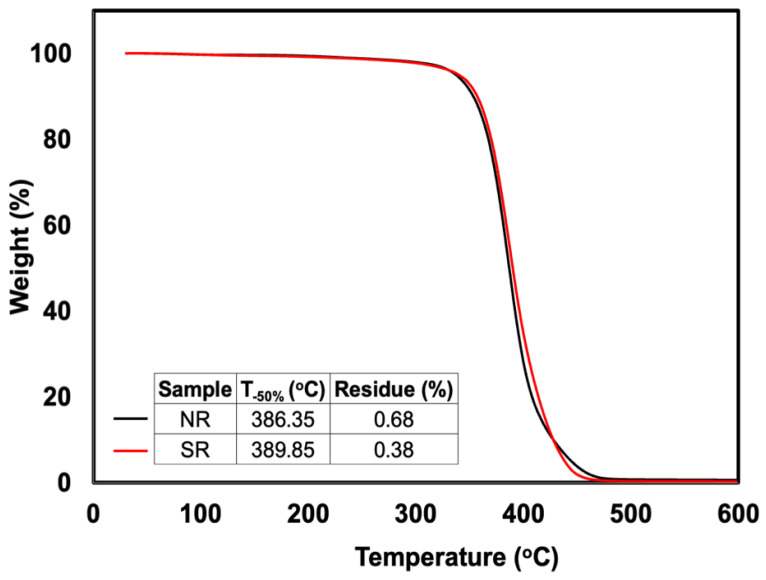
TG curves of NR and SR.

The change in a sample’s mass as a function of temperature in a controlled atmosphere is measured by thermogravimetric analysis (TGA). The measurement is primarily used to ascertain the compositional characteristics and thermal and oxidative stabilities of materials. The thermal decomposition behavior of raw NR and SR is shown in Figure 2. The decomposition temperature at 50% weight loss and char residue are also listed and embedded in this Figure. Notably, the decomposition temperature at 50% weight loss of SR was higher than NR. This is simply due to the higher thermal stability of SR as the grafting of MMA onto NR reduces the diene content in the NR. The thermal stability of SR was then improved. Moreover, both NR and SR exhibited low residue which was less than 1%.

### 3.2. Cure Characteristics

**Figure 3:**
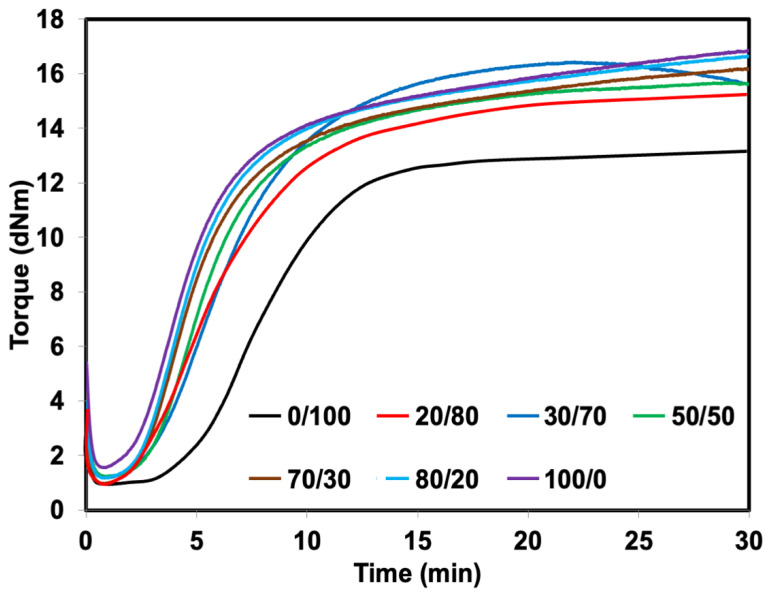
Rheometric curves of SR/NR blends.

The rheometric curves of the foams are shown in Figure 3. All the curves showed a marching trend except for the blend at 30/70 phr/phr. The marching curve is present when there is a development of crosslinking [20]. The reversion trend observed for the blend at 30/70 phr/phr may be due to the SR itself. The received SR was from the waste of NR-g-PMMA production, which is uncontrollable material. This phenomenon also occurred in the experiment by Nakason et al. [18], who varied accelerator types to the NR-g-PMMA. They found the reversion of the rubber vulcanizates regardless of accelerator types. The reversion may be associated with the degradation of NR molecules. Separately, the important point to highlight is the increment of the minimum torque (M_L_) and maximum torque (M_H_) after replacing NR with SR, indicating that the material became stiff over the addition of SR. Grafting MMA onto NR makes the rubber harder due to the presence of the PMMA component as a glassy thermoplastic phase. The scorch time (t_S1_) and curing time (t_C90_) were reduced with the addition of SR. t_S1_ is the induction time experienced by a rubber compound before vulcanization is initiated. At the same time, t_C90_ is the time rubber takes to become 90% vulcanized. The decrease in these two values indicated that SR could quicken the vulcanization time of rubber.

According to Harpell et al. [21] and Bhatti et al. [22], the decomposition of ADC produces hydrazodicarbonamide, urazol, and a gaseous mixture of nitrogen (N_2_), carbon monoxide (CO), cyanic acid (HNCO), and ammonia (NH_3_) through competitive and exothermic chemical pathways. N_2_ is the primary source of gas that makes the foam free-flowing. Depending on the circumstances of the process and the status of the result, some paths may be preferred over others. The focal point here is the production of ammonia, which tends to react with PMMA through ammonolysis and results in the formation of primary amide. Such amide derivative may cause the vulcanization of rubber to accelerate, hence decreasing the ts_1_ and tc_90_ in the blends containing a higher content of SR. On the contrary, this kind of phenomenon did not happen for un-foamed specimens. As reported by Nakason et al. [18], they found that the addition of NR-g-PMMA prolongs the ts_1_ and tc_90_. This was attributed to the polar functional groups of the graft copolymer absorbing certain accelerators as a result of their polarity. Consequently, the accelerator in these quantities was unable to accelerate the vulcanization process. Therefore, a longer cure period was needed to finish the crosslinking process and get the optimum curing capabilities.

### 3.3. Physical Properties, Appearance, and Morphologies

Table 3 lists the relative foam densities and expansion ratios of the foams with different ratios of blending. As a higher content of SR was used, less gas was subsequently generated due to less flexibility of SR. This increased the relative foam density, which significantly increased from 0.57 g/cm^3^ to 0.84 g/cm^3^. Higher SR content hardens the rubber matrix, thus restricting the escape of gas through the foam surface. This allowed the foam to have less expansion and, consequently, produce foam with a higher relative density [23]. The relative foam density was directly related to the porosity values and expansion ratio. For this reason, the increased relative foam density when using a high content of SR revealed less expansion of these specimens. The porosity values and expansion ratios are also shown in Table 3. The increase in relative foam density also played a role by decreasing the size of cells per unit volume. The glassy phase of SR may prevent the penetration of gas during compression. Table 3 also lists the hardness of the foams prepared using various SR content. The hardness increased with an increase in the SR content. This was due to the higher foam porosity in the matrix during the formation of the gas phase. Again, an inherent chain stiffness of SR responds to an increase in the hardness of the samples.

**Figure 4:**
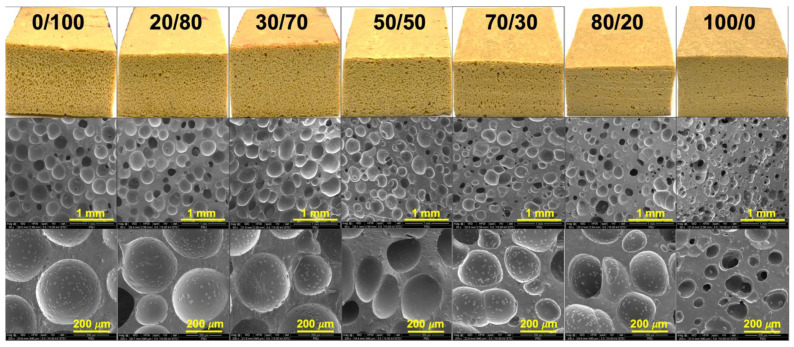
SEM images obtained from the samples’ surface of SR/NR blends.

Further evidence can be identified from the SEM images shown in Figure 4. The SEM images showed a systematic correlation between the number of cells per unit volume and the average cell size. An increase in the SR resulted in smaller cells and a thick cell wall, indicating the difficulty of gas to diffuse and generate the foams. In this experiment, ADC content was fixed at 5 phr, and the volume of gas generated after the decomposition was assumed to be the same. However, the gas produced could not diffuse or penetrate through the rubber matrix, especially at a higher content of SR. Consequently, the number of cells per unit volume decreased, resulting in a smaller average cell size and a thicker cell wall in the foam [9]. Increasing the levels of SR also affected cell distribution. The foam cell distributed unevenly as the content of SR increased, and random cell size was seen for the sample using a high content of SR. The SEM images are in good agreement with the porosity values and expansion ratios reported in the previous section.

### 3.4. Oil Absorbency

**Figure 5:**
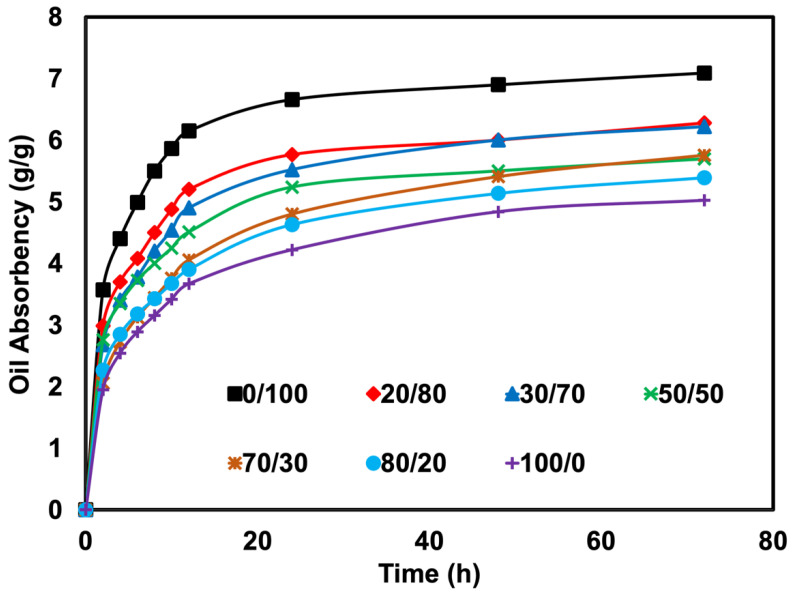
Oil absorbency as a function of contact time of SR/NR blends.

**Figure 6:**
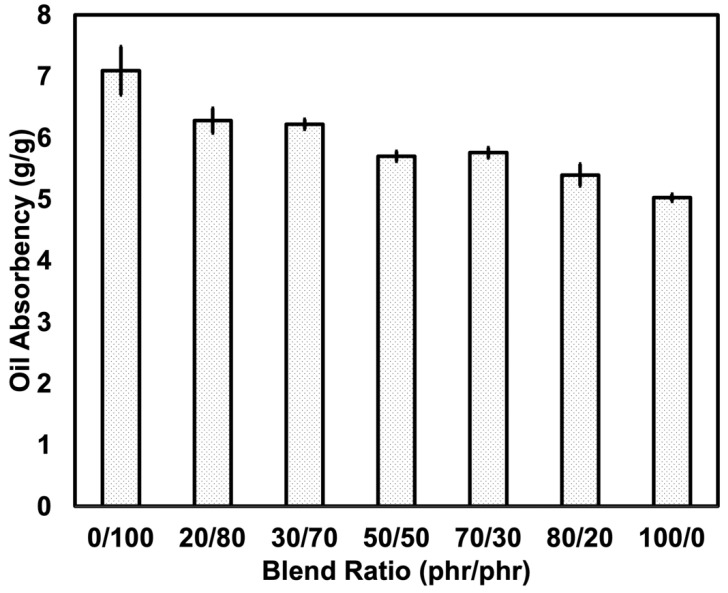
Oil absorbency of SR/NR blends at 72 h uptake.

**Figure 7:**
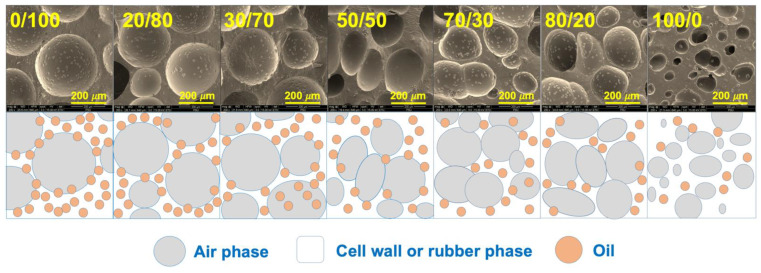
Swelling schematic of oil penetrant through the SR/NR blends.

Figure 5 and Figure 6 depict the oil absorbency over the contact time and the equilibrium absorbency of an oil-absorbent material prepared from various blend ratios. It was observed that the oil absorbency was reduced over the content of SR. Adding SR reduced the oil swellability from 7.09 g/g to 5.02 g/g. The results obtained in this work were found differently compared to previous literature [7,8]. Previous works prepared the foam differently, where the NR and NR-g-PMMA were mixed in the latex stage. The generation of the foam or foam forming was done by Dunlop Process [24]. The foam is generated easier than with the dry rubber method. This has provided a different cellular structure. In this experiment, foaming became more difficult due to the harder phase of SR, where the decomposed gas ineffectively penetrated throughout the matrix. This can be seen from the morphology of the foams (see Figure 4). As mentioned previously, the size of cells decreased, and the thickness of cell walls increased when a higher content of SR was used. As the cell size decreased, the porosity decreased, and the oil could not penetrate easily from one cell to another, leading eventually to a decrease in the swelling uptake. This is further explained in a study by Lee et al. [25], which stated that the swelling of NR foam is influenced by the cell structure and the density, where a lower foam density has caused a higher swelling uptake. This explanation can be clearly understood when correlated with the swelling schematic shown in Figure 7.

### 3.5. Diffusion Study

**Figure 8:**
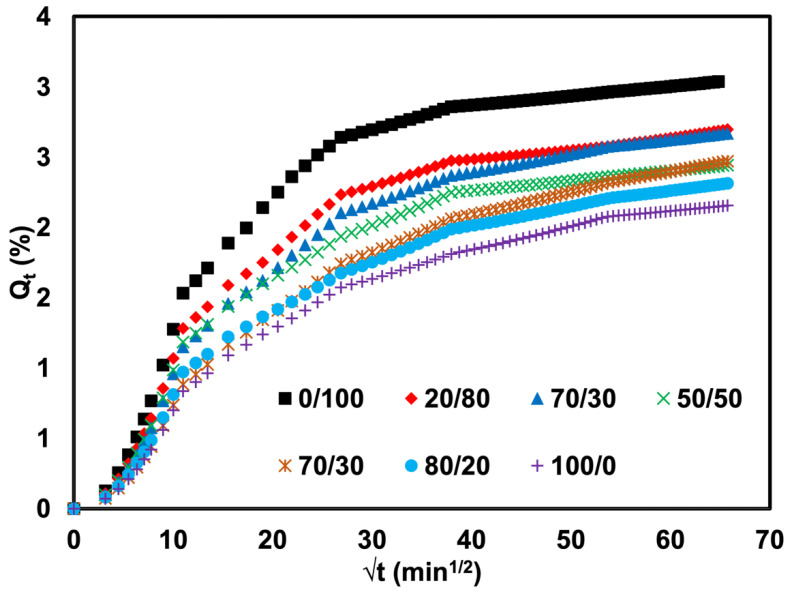
Mole percent uptake as a function of time of SR/NR blends.

**Figure 9:**
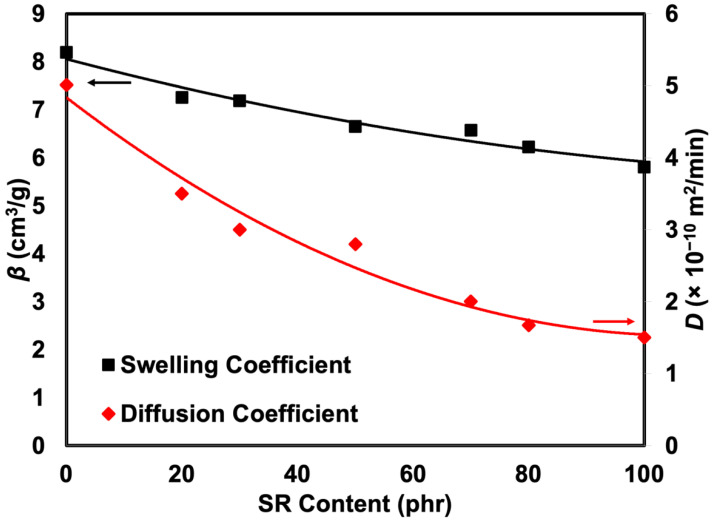
Swelling and diffusion coefficients of SR/NR blends.

The most common method of delivering small compounds to polymers is solution diffusion. The penetrant molecules, in this case crude oil, are first absorbed by the rubber before being diffused through it. The sorption data of the crude oil into the rubber at room temperature was determined and expressed as the mole percent uptake (*Q_t_*) against *t*^1/2^ (min^1/2^), as shown in Figure 8. The curves show gradual steps of absorption. A significant concentration gradient caused the initial steep zone with a high sorption rate. In contrast, as equilibrium approached, the sorption rate decreased in the later regions. It is well acknowledged that the cross-link density of a network chain and the equilibrium mole percent uptake are correlated [26] and the efficacy of oil or solvent in penetrating rubber molecules since this study was prepared under controlled formulation. It was expected that the cross-linking was more or less the same. The only reason to be regarded with a lower mole percent uptake or penetration into the rubber was due to the efficacy of oil or solvent to penetrate. Thus, the swelling resistance of the blends containing a higher content of SR increased. Similar results were obtained for the rubber’s diffusion and swelling coefficients, as shown in Figure 9. As the SR component increased from 0 to 100 phr, the swelling coefficient of the rubber steadily dropped from 8.19 to 5.81 cm^3^/g. Therefore, it was more difficult for the oil to penetrate the rubber.

**Figure 10:**
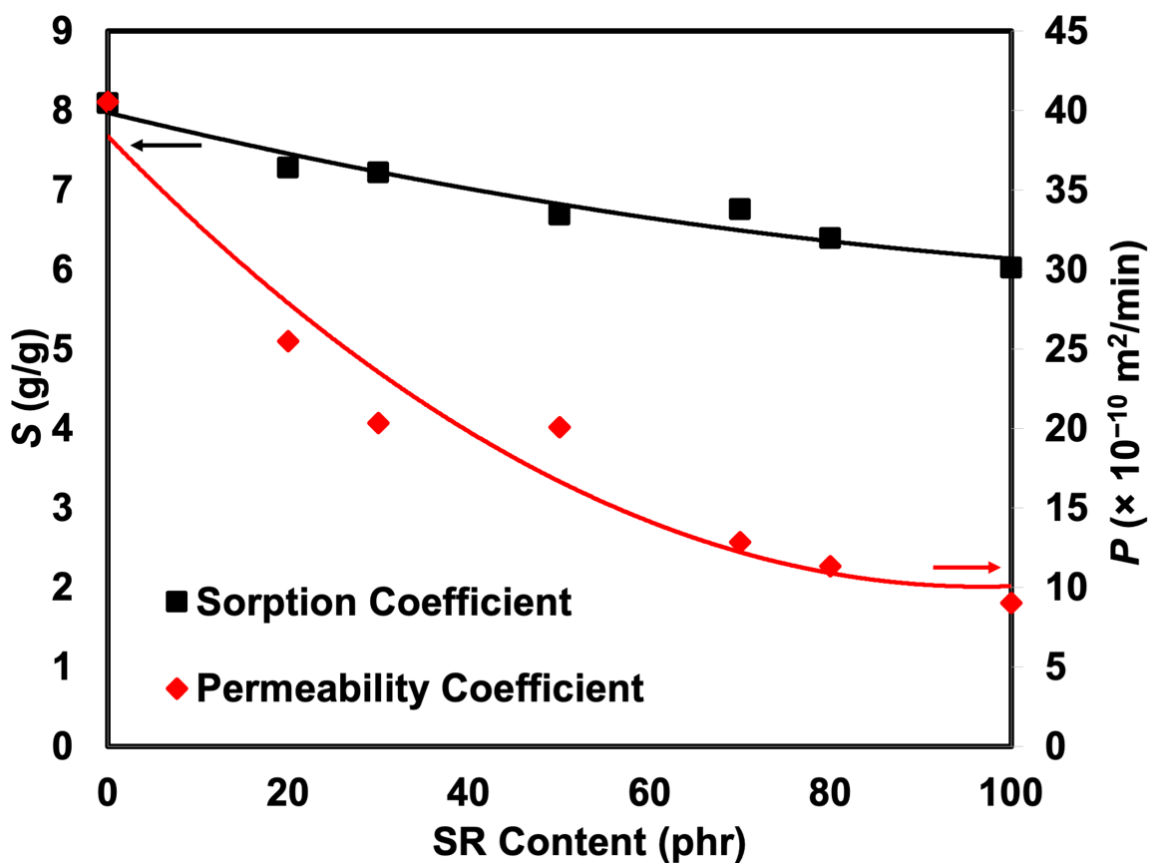
Sorption and permeability coefficients of SR/NR blends.

The diffusion coefficient (*D*) is a kinetic parameter that relies on segmental mobility. Based on the outcome depicted in Figure 9, *D* was determined using an equation derived from the second Fickian’s law. It demonstrates a non-steady decreasing trend, indicating that the oil had difficulty penetrating the rubber matrix with a high SR content. The sorption coefficient and the permeability coefficient are two more characteristics that can be derived from the rubber’s diffusion studies (see Figure 10). The initial absorption and dispersion of permeate molecules into the rubber matrix can be explained by the sorption coefficient. In contrast, the amount of penetrant that passes through a consistent area of the sample per minute is shown by the permeability coefficient. As the SR content increased, the rubber’s sorption and permeability coefficients gradually declined, indicating that SR segments impede oil diffusion of the rubber foam upon the addition of SR.

### 3.6. Transport Mechanism

**Figure 11:**
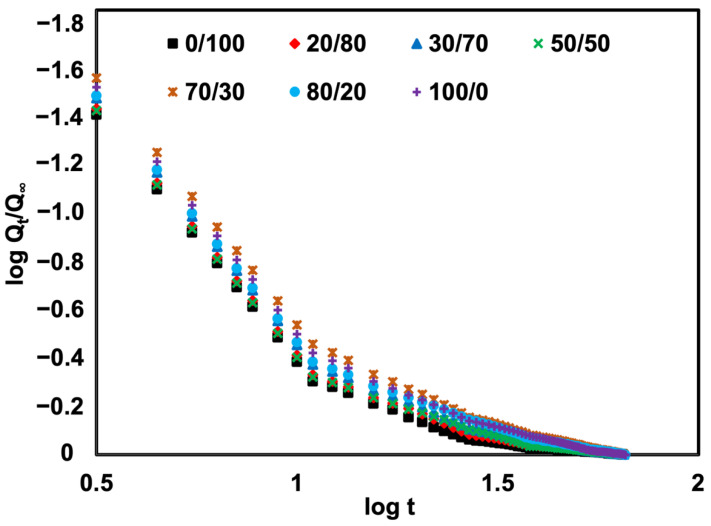
Fitted sorption curves of SR/NR blends.

Figure 11 displays the results of fitting the oil uptake data into Equation (10) to determine the mode of the transport mechanism. According to Table 4, linear regression analysis of the initial linear slope was used to obtain the values of *n* and *k*. Based on the relative mobility of the penetrant and polymer segments, a few categories can be used to classify the transport mechanism, which are: (i) Case I or Fickian diffusion, (ii) Case II diffusion, and (iii) non-Fickian or anomalous diffusion [27]. When *n* has a value of less than or equal to 0.5, the concentration gradient acts as the main driving factor for diffusion in a Fickian transport mode. The diffusion rate is, therefore, lower than the polymer chain relaxation rate. However, for Case II transport, where *n* is equal to 1, the diffusion rate is greater than the relaxation process. On the other hand, if the *n* value is between 0.5 and 1, the transport is anomalous, and the diffusion rate matches the rate at which the polymer chains are relaxing [28].

It is clear from Table 4 that the transport mechanism was anomalous for rubber containing SR between 0 and 100 phr. This result agrees well with the sorption curve in Figure 8, which, over the same time period, showed a modest increase in the mole percent solvent uptake before reaching equilibrium. Polymer chains adapt to a penetrant’s presence quickly, but it takes a while for the equilibrium solvent absorption to occur. Additionally, the value of *k* reflects how oil penetrates rubber or how the penetrant interacts with the rubber matrix. A lower *k* value represents a lower speed of solvent or oil-penetrating rubber. Here, the calculated results show that the penetrant was more difficult when having more SR. This is simply because the foam contains a thicker cell wall which deactivates the efficacy of oil in penetrating rubber molecules.

### 3.7. Thermal Stability

**Figure 12:**
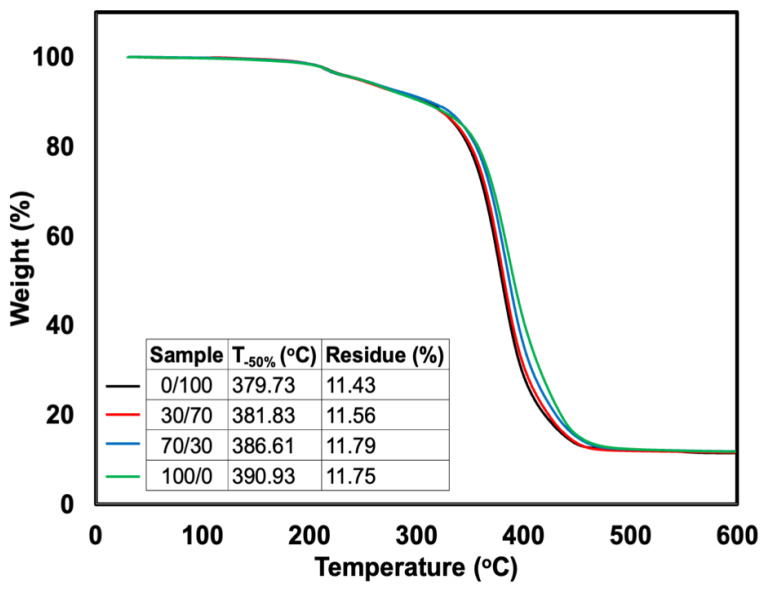
TG curves of SR/NR blends.

Figure 12 depicts the TG curves of the samples. The decomposition temperature at 50% weight loss (T_−50%_) and the content of char residue are embedded in this Figure. Two regions of degradation of specimens were seen. The initial minor mass loss at around 180–200 °C was due to the presence of volatile matter such as stearic acid and TDAE oil, and the process was complete at about 300 °C [29]. Then, the major step of degradation of the blends (330–450 °C) was caused by the degradation of both SR and NR segments. It is noteworthy that the specimens’ thermal stability marginally improved as the content of SR increased. This can be seen from the T_−50%_, which clearly shows that by introducing SR, the temperature was shifted to a higher temperature. The enhancement in thermal stability can be evidently related to the original stability of SR seen previously in Figure 2. The replacement of MMA onto the backbone of NR has made the NR-g-PMMA more stable against the formation of degradation. A similar observation was also found in the literature [17]. They found that increasing the content of MMA onto NR has enhanced the thermal resistance of rubber. With increased PMMA content in the grafting reaction, stronger chemical interactions between the molecules were speculated to be the cause. Additionally, increasing oxygen compounds exhibited a higher resistance to thermal degradation than that of the original NR. Moreover, the decomposition temperature of foam specimens was slightly lower than raw rubbers (see Figure 2). This is because the foam was heated during processing and vulcanization. Therefore, slight degradation of foam specimens may occur during such a process.

## 4. Conclusions

The aim of this work was to utilize the waste from the production of NR-g-PMMA as an oil-absorbent rubber. The rubber blends were then turned into cellular structures to increase the oil swellability. Results indicated that adding SR quickened the vulcanization process of rubber due to the ammonolysis taking place during the vulcanization of the foam. It was observed that the cellular structure of the foam was difficult to generate when using a higher content of SR. This made the oil swellability reduce from 7.09 g/g to 5.02 g/g. This agreed well with the study of swelling kinetics. It demonstrates a non-steady decreasing trend; the transport mechanism was anomalous for rubber containing SR between 0 and 100 phr. There was a minor degradation of the samples at temperatures of 180–300 °C due to the presence of volatile matters, mainly from TDAE oil. This would not affect the application of oil-absorbent rubber since it was added to facilitate the foaming process. However, it is interesting to note that the prepared oil-absorbent rubber still provided higher thermal stability, particularly at temperatures over 400 °C. This is a very good compromise between oil absorbency and thermal stability. Based on the overall properties, the SR content at 30 phr is suggested. This is considered from oil absorption capacity, yet other factors include blend processability, foam structure, and physical and thermal properties. The results obtained from this study will be used for further experiments on the enhancement of oil absorbency by applying other key factors. This work is a good initiative for preparing the oil-absorbent material based on scrap from modified natural rubber production. At the same time, our work provides an alternative route to fabricate oil-absorbent foam for an oil recovery application.

## Figures and Tables

**Table 1:** Characteristics of NR and SR.

Characteristics	Values	
	NR	SR
Ash content	0.02%	0.04%
Volatile matter	0.52%	0.67%
Acetone extract	2.67%	14.99%
Soluble fraction	98.7%	96.5%
Nitrogen content	0.26%	Not detected
Original plasticity (P_o_)	40.3	72.1
Moony viscosity (ML 1 + 4 at 100 °C)	77.4	113.1

**Table 2:** Mixing sequence and compounding ingredients used for preparing the rubber compounds.

Mixing Sequence	Ingredients	Amount (phr)	Mixing Time (min)
1	SR/NR *	100	5
2	Stearic acid	1	1
3	TMQ	1	1
4	ZnO	5	1
5	CaCO_3_	10	3
6	TDAE oil	10	2
7	CBS	2.5	1
8	ADC	5	1.5
9	Sulfur	0.5	1

* The amount of SR and NR were varied.

**Table 3:** Physical properties of SR/NR blends.

SR/NR (phr/phr)	Relative Foam Density (RFD)	Expansion Ratio	Porosity (1 − RFD)	Hardness (Shore OO)
0/100	0.57 ± 0.01	1.75	0.43	58 ± 0.71
20/80	0.59 ± 0.02	1.70	0.41	61 ± 0.44
30/70	0.66 ± 0.01	1.52	0.34	64 ± 0.50
50/50	0.70 ± 0.01	1.44	0.30	69 ± 0.26
70/30	0.72 ± 0.02	1.38	0.28	81 ±0.56
80/20	0.75 ± 0.01	1.34	0.25	86 ± 0.52
100/0	0.84 ± 0.01	1.19	0.16	91 ± 0.59

**Table 4:** The values of *n* and *k* of SR/NR blends.

SR/NR (phr/phr)	*n*	*k*
0/100	0.7348	0.0575
20/80	0.7566	0.0517
30/70	0.7587	0.0512
50/50	0.8074	0.0420
70/30	0.8178	0.0394
80/20	0.8524	0.0344
100/0	0.8668	0.0297

## Data Availability

The data presented in this study are available on request from the corresponding author.
